# Effect of mRNA Vaccines in Preventing COVID-19 Severe Pneumonia Among COVID-19 Patients in Japan

**DOI:** 10.2188/jea.JE20210487

**Published:** 2022-03-05

**Authors:** Rumi Matsuo, Naomi Matsumoto, Tomoka Kadowaki, Toshiharu Mitsuhashi, Soshi Takao, Takashi Yorifuji

**Affiliations:** Department of Epidemiology, Graduate School of Medicine, Dentistry and Pharmaceutical Sciences, Okayama University, Okayama, Japan

The emergence of coronavirus disease 2019 (COVID-19) has catalyzed a new phase in vaccine development.^1^ Studies of the effectiveness of mRNA vaccines against COVID-19 have been conducted mainly in Western countries, and data from Asia are comparatively limited. Moreover, previous studies examined the effect of mRNA vaccines among uninfected individuals, but few studies have focused on severe outcomes among diagnosed patients. We examined the effect of mRNA vaccines in preventing COVID-19-related severe outcomes among patients in Japan.

The study population were patients with COVID-19 aged 20 years and older reported to the Okayama City Public Health Center in Okayama City with symptom onset between July 1 and September 30, 2021. All patients were diagnosed with COVID-19 using polymerase chain reaction or antigen testing. For asymptomatic patients, the date of examination was used as the onset date. All cases occurring in Okayama City were reported to the center, and more than 90% of cases involved infection with the delta variant during the study period.^2^ Among the 3,616 cases reported to the center, we excluded teenagers and younger children because of low vaccination coverage and low risk of severe disease, leaving 2,882 adult patients. We assessed COVID-19 severity in these patients until October 14, 2021.

The Public Health Center confirmed the vaccination status of patients and assessed COVID-19 severity in all patients and confirmed with the hospital when patients were hospitalized. We excluded a total of 174 patients with unknown vaccination status and 16 patients with unknown COVID-19 severity. Ultimately, a total of 2,692 patients remained in the final analysis.

We classified the patients into three vaccination categories: fully vaccinated (≥14 days after the second dose), partially vaccinated (received only the first dose or <14 days after the second dose), or unvaccinated prior to symptom onset. During the study period, the only vaccines available in Okayama were the mRNA vaccines BNT162b2 and mRNA-1273.

In keeping with the guidelines for COVID-19 in Japan,^3^ we defined the primary outcome of our study as COVID-19 severe pneumonia with respiratory failure (eg, oxygen saturation [Sp0_2_] on room air ≤93%, requiring supplemental oxygen), intensive care unit admission, mechanical intubation, and/or death. The secondary outcome was COVID-19 pneumonia diagnosed via imaging.

Following descriptive analyses, we conducted Poisson regression with robust error variance to examine the associations between vaccination status and the primary and secondary outcomes. We estimated risk ratios (RRs) and their 95% confidence intervals (CIs) adjusting for age category, sex, smoking status, and comorbidities based on the results of a previous study.^4^ The study was approved by the Okayama University Graduate School of Medicine, Dentistry and Pharmaceutical Sciences Ethical Committee (No. K2110-020), which waived requirement for informed consent because this was a retrospective study and complete anonymity was ensured.

More than half of study participants were aged 20 to 39 years (60.62%; *n* = 1,632) and men (56.35%; *n* = 1,517). Compared with unvaccinated patients, the adjusted RRs for COVID-19 severe pneumonia were 0.53 (95% CI, 0.30–0.94) for partially vaccinated and 0.25 (95% CI, 0.09–0.67) for fully vaccinated patients (Table 1). Although the results of stratified analyses were unstable, the preventive effects of vaccination were observed even in older patients and in patients with comorbidities.

**Table 1.** Effect of mRNA vaccines in preventing COVID-19 severe pneumonia among COVID-19 patients in Japan

	Primary outcome COVID-19 severe pneumonia		Secondary outcome COVID-19 pneumonia	
	
	case/Total (%)	Adjusted RR (95% CI)^a^	case/Total (%)	Adjusted RR (95% CI)^a^
Overall				
Unvaccinated	95/2,252 (4.2)	1.0 (reference)	185/2,252 (8.2)	1.0 (reference)
Partially vaccinated	15/291 (5.2)	0.53 (0.30–0.94)	28/291 (9.6)	0.53 (0.35–0.79)
Fully vaccinated	4/149 (2.7)	0.25 (0.09–0.67)	8/149 (5.4)	0.25 (0.13–0.50)
Stratified analysis				
Age group				
<50 years old				
Unvaccinated	55/1,915 (2.9)	1.0 (ref)	109/1,915 (5.7)	1.0 (ref)
Partially vaccinated	4/165 (2.4)	0.51 (0.16–1.61)	9/165 (5.5)	0.67 (0.33–1.37)
Fully vaccinated	0/63 (0.0)	NA	0/63 (0.0)	NA
≥50 years old				
Unvaccinated	40/337 (11.9)	1.0 (ref)	76/337 (22.6)	1.0 (ref)
Partially vaccinated	11/126 (8.7)	0.54 (0.29–1.03)	19/126 (15.1)	0.49 (0.31–0.80)
Fully vaccinated	4/86 (4.7)	0.33 (0.12–0.91)	8/86 (9.3)	0.36 (0.18–0.72)
Comorbidity^b^				
None				
Unvaccinated	30/1,474 (2.0)	1.0 (ref)	71/1,474 (4.8)	1.0 (ref)
Partially vaccinated	3/140 (2.1)	0.60 (0.18–2.06)	5/140 (3.6)	0.44 (0.18–1.10)
Fully vaccinated	1/74 (1.4)	0.31 (0.05–2.06)	3/74 (4.1)	0.40 (0.14–1.14)
More than one				
Unvaccinated	63/731 (8.6)	1.0 (ref)	113/731 (15.5)	1.0 (ref)
Partially vaccinated	10/145 (6.9)	0.58 (0.30–1.11)	19/145 (13.1)	0.62 (0.40–0.97)
Fully vaccinated	3/72 (4.2)	0.26 (0.09–0.76)	5/72 (6.9)	0.21 (0.10–0.47)

BMI, body mass index; CI, confidence interval; COVID-19, coronavirus disease 2019; NA, not available; RR, risk ratio.

^a^Adjusted for age category (20–39, 40–59, 60–79, and ≥80 years), sex, smoking status, hypertension, diabetes, heart disease, respiratory disease, and obesity (BMI ≥25 kg/m^2^). A total of 56 participants had missing BMI information.

^b^Comorbidities: hypertension, diabetes, heart disease, respiratory disease, and obesity. RRs were estimated by adjusting for age category, sex, and smoking status. A total of 56 participants had missing BMI information.

We demonstrated the effect of mRNA vaccines (BNT162b2 and mRNA-1273) in protecting against COVID-19 severe pneumonia and pneumonia among patients with COVID-19 in Japan. Our results are consistent with those of previous studies of healthy individuals in Western countries.^5^ We provide further evidence that mRNA vaccines can decrease the risk of severe outcomes by 70% among patients with COVID-19, consistent with a study from England.^6^

A strength of our study was that we used data from a Public Health Center and all cases occurring in a whole city were included. A limitation of our study was that information was collected by the patients, so there is a possibility of misclassification. Although we adjusted for several risk factors for severity, which would be related to the vaccination status in the statistical analyses, there is a possibility of residual confounding. Moreover, vaccination coverage is still limited, especially for patients <50 years old, so we could not evaluate the effects among that age category.
